# Patient comorbidities, medication intake, and mortality in revision surgery for periprosthetic joint infection of the hip and knee: analysis of 346 patients

**DOI:** 10.1186/s13018-025-06209-w

**Published:** 2026-03-03

**Authors:** Filippo Migliorini, Christian David Weber, Andreas Bell, Marcel Betsch, Nicola Maffulli, Vanessa Poth, Michael Celik, Tommaso Bardazzi, Ulf Krister Hofmann, Frank Hildebrand, Arne Driessen

**Affiliations:** 1 Department of Trauma and Reconstructive Surgery, University Hospital of Halle, Martin-Luther University Halle-Wittenberg, Ernst-Grube-Street 40, Halle (Saale), 06097 Germany; 2 Department of Orthopaedics and Trauma Surgery, Academic Hospital of Bolzano (SABES-ASDAA), Bolzano, 39100 Italy; 3 Department of Life Sciences, Health, and Health Professions, Link Campus University, Rome, Italy; 4 Department of Orthopaedic, Trauma, and Reconstructive Surgery, RWTH University Hospital, Aachen, 52074 Germany; 5 Department of Orthopaedic and Trauma Surgery, Eifelklinik St.Brigida, Kammerbruschstr. 8, Simmerath, 52152 Germany; 6 Department of Orthopaedic and Trauma Surgery, University Hospital of Erlangen, Erlangen, 91054 Germany; 7 Faculty of Medicine and Psychology, University “La Sapienza” of Rome, Rome, Italy; 8 School of Pharmacy and Bioengineering, Faculty of Medicine, Keele University, Stoke On Trent, ST4 7QB UK; 9 Centre for Sports and Exercise Medicine, Barts and the London School of Medicine and Dentistry, Mile End Hospital, Queen Mary University of London, London, E1 4DG UK; 10 Department of Anesthesiology, Eifelklinik St. Brigida, Simmerath, Germany; 11 Department of Orthopaedic and Trauma Surgery, Luisen Hospital, Aachen, Germany

**Keywords:** Arthroplasty, Comorbidities, Periprosthetic joint infection, Mortality, Knee, Hip, Infection

## Abstract

**Background:**

Patient comorbidities and medication intake impact on the mortality rate in revision surgery for periprosthetic joint infection (PJI) of the lower limb. The present study collected data from patients who underwent revision surgery for PJI of total hip arthroplasty (THA) or total knee arthroplasty (TKA). Data regarding comorbidities and medication intake for each patient were collected to investigate whether comorbidities and medication intake influence in-hospital mortality in patients who underwent revision surgery for PJI of a THA or TKA.

**Methods:**

The present study follows the STROBE Statement. Our institutional databases were searched using the OPS (operation and procedure codes) 5–823 and 5–821 in combination with the ICD (International Statistical Classification of Diseases and Related Health Problems) codes T84.5, T84.7 or T84.8. All patients with hip or knee implant infections who underwent revision surgery were retrospectively retrieved and included in the present study.

**Results:**

Data from 346 patients were collected (181 THAs and 165 TKAs). Patients with renal insufficiency demonstrated a statistically significant greater risk of in-hospital mortality (95% CI 0.0131 to 0.1132), as did patients with a history of malignancy (95% CI 0.1478 to 0.7497), and patients with dementia (95% CI 0.0398 to 0.3791). Nicotine and alcohol abuse, diabetes mellitus, arterial hypertension, hereditary thrombophilia, hereditary haemorrhages, cerebrovascular diseases, coronary heart diseases, chronic obstructive pulmonary disease osteoporosis, liver cirrhosis, rheumatoid arthritis, acute dental infection did not influence in the in-hospital mortality rate in patients who underwent revision surgery for PJI of a THA or TKA. Patient medication therapy did not impact the risk of in-hospital mortality in PJI.

**Conclusion:**

Patients undergoing revision surgery for PJI after total hip and knee arthroplasty show an increased in-hospital mortality in the presence of the following comorbidities: dementia, renal insufficiency, and history of malignancy. Based on the present results, further infection prevention and geriatric co-management strategies should be evaluated for patients undergoing revision arthroplasty of the hip and knee for PJI.

## Introduction

Total hip arthroplasty (THA) and total knee arthroplasty (TKA) reduce pain and improve patients' quality of life [1, 2]. Joint arthroplasty is cost-effective, especially in the elderly population [3, 4]. Given the satisfying surgical outcomes, the increasing population age, and easier access to health care, the number of arthroplasties performed every year is increasing. In Germany, 233,424 total hip arthroplasties (THAs) and 187,319 total knee arthroplasties (TKAs) were performed in 2016 [5]. As a direct consequence of the increasing trend in the number of arthroplasties per year, the number of revisions is increasing. From 2016 to 2019, revisions of THAs and TKA increased by 1% (35,464 to 35,859) and 4% (24,940 to 25,841), respectively [6, 7]. The five-year survival rate is approximately 81.0% and 87.4% in revision THA and TKA, respectively [8]. A third of all revisions are performed because of periprosthetic joint infections (PJI) [5]. Posttraumatic osteoarthritis, chronic liver disease, obesity, immunosuppressive therapy, and infections in other sites increase the risk of developing PJI [9–12]. Obesity is associated with an increased risk of infection up to four folds [13]. Diabetes mellitus doubles the risk of surgical infections compared to healthy control [14]. Previous surgeries, prolonged operating time, or inadequate antibiotic prophylaxis may also predispose to infection [15]. Revision of an infected arthroplasty may represent a major physical challenge, especially in the elderly, who present greater comorbidities and medication intake compared to younger populations. Whether comorbidities and medication intake impact the risk of mortality following surgical revision for periprosthetic infections is unclear. Although certain conditions negatively impact the risk of PJI, it is not clear to which extent they influence the rate of mortality in revision settings for THA and TKA. High quality clinical investigations involving large samples which evaluate the impact of patient comorbidities and medication intake on the mortality rate in revision surgery for PJI are limited. The present study collected data from patients who underwent revision surgery for PJI of THA or TKA. Data regarding comorbidities and medication intake for each patient were collected. This study investigated whether comorbidities and medication intake exert an influence on mortality in patients who underwent revision surgery for PJI of THA or TKA.

## Methods

### Study design

The present study was conducted according to the principles of the Declaration of Helsinki and was approved by the ethics committee of the RWTH Aachen University (project ID EK 121/22). The present study follows the Strengthening the Reporting of Observational Studies in Epidemiology: the STROBE Statement [16]. The present investigation was conducted at the Department of Orthopaedics, Trauma and Reconstructive Surgery, of the University Hospital RWTH Aachen, Germany, the Department of Orthopaedics of the Eifelklinik St. Brigida of Simmerath, Germany, and the Department of Orthopaedics of the University Hospital of Salerno, Italy. In August 2022 the clinical databases of these three institutions were accessed with no time constraints. The database of the University Hospital of Salerno was screened by hand by two authors. For the databases of the German institutions, the OPS (operation and procedure codes) 5–823 and 5–821 were used in combination with the ICD (International Statistical Classification of Diseases and Related Health Problems) codes T84.5, T84.7 or T84.8 (Table 1). Patient data were included in a Microsoft Excel spreadsheet (version 16.6).

**Table 1:** ICD codes used for the database search

Code	Diagnosis/Procedure
5–823	Revision, replacement and removal of a knee joint endoprosthesis
5–821	Revision, replacement and removal of a hip joint endoprosthesis
T84.5	Infections and inflammatory reactions caused by a joint endoprosthesis
T84.7	Infection and inflammatory reaction from other orthopaedic endoprostheses, implants or transplants
T84.8	Other complications from orthopaedic endoprostheses, implants or transplants

All patients with hip or knee PJI who had undergone THA or TKA revision surgery were retrieved. The inclusion criteria were: arthroplasty of knee or hip; microbiological evidence of pathogen using joint aspiration and/ or intraoperative histologic examination and/ or of blood cultures; presence of at least one of these signs of inflammation at the joint: heat (calor), pain (dolor), redness (rubor), and swelling (tumor). The exclusion criteria were: any other non-infective ailment in a previously implanted arthroplasty; arthroplasty performed in joints other than knee and hip.

### Data collection

The following data was recorded: gender, age at admission, height, weight and BMI, side, joint and the year of implantation. Data concerning the number and the length of the hospitalization, and the number of revisions were collected. Data on mortality was also retrieved. The perioperative risk was assessed using the American Society of Anaesthesiologists (ASA) as ASA 1 to ASA 6 [17]. The comorbidities included for analysis were nicotine and alcohol abuse, renal insufficiency, diabetes mellitus, arterial hypertension, hereditary thrombophilia, hereditary haemorrhages, cerebrovascular diseases, coronary heart diseases, history of malignancies, dementia, chronic obstructive pulmonary disease osteoporosis, liver cirrhosis, rheumatoid arthritis, and acute dental infection. A creatinine value > 1.2 mg/dl was considered to indicate kidney failure. We did not classify diabetic patients as having diabetes mellitus type I or Type II. Moreover, we collected data on the following medications: coumarin, novel oral anticoagulants (NOACs), heparins, angiotensin-converting enzyme (ACE) inhibitors, corticosteroids, angiotensin-II-receptor antagonists.

### Statistical analysis

The evaluation was conducted by the main author (FM) using the IBM SPSS Statistics software package, version 25. For descriptive statistics, the frequency (amount of events/number of observations) was used for binary data. Arithmetic mean and standard deviation were used for continuous data. To evaluate survivorship and the risk of mortality according to patient comorbidities and medications, the odd ratio (OR) effect measure was evaluated. The confidence intervals (CI) was set at 0.95. A standard normal deviation (z-value) was calculated as ln(OR)/SE{ln(OR)}, and the *P*-value is the area of the normal distribution that falls outside ± z. Values of *P* < 0.05 were considered statistically significant.

## Results

### Patient recruitment

**Fig. 1:**
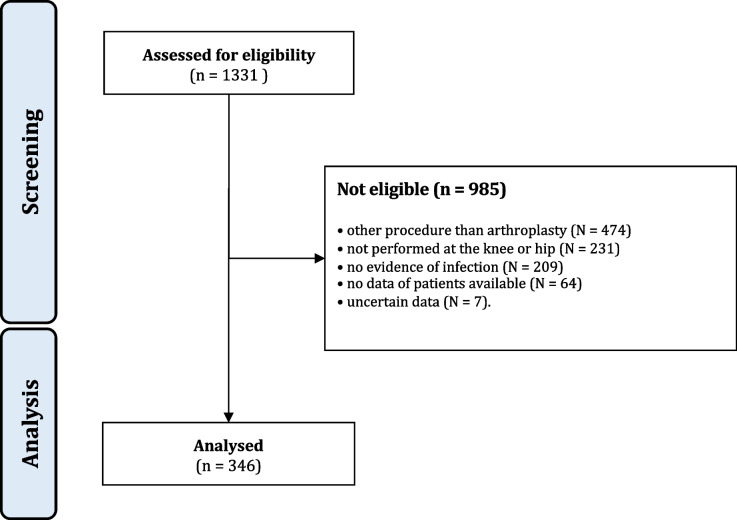
STROBE diagram of the patient recruitment

The database search resulted in 1331 procedures. Of them, 985 procedures were excluded with reason: procedure other than revision arthroplasty (*N* = 474), not performed at the knee or hip (*N* = 231), no evidence of infection (*N* = 209), no data of patients available (*N* = 64), uncertain data (*N* = 7). Finally, 346 patients were considered in the present study (Fig. 1).

### Patient demographic

Data from 346 patients were collected (181 THAs and 165 TKAs). 44% (152 of 346 patients) were women. Overall, the mean age was 67.8 years, and the mean BMI was 29.2 kg/m^2^. The mean hospitalization length was 23.5 days. 38% (132 of 346) of patients presented a re-infection of a previously treated PJI. Demographic information of the patients is shown in Table 2. Liners were changed in 171 patients; implants were removed in 155 patients. Amputation was necessary for two patients. The pathogen was detected in 37% (128 of 346) of joint aspirations, 85% (294 of 346) of intraoperative microbiologic examinations, and 17% (59 of 346) of blood cultures. 312 (90%) patients survived and 34 (10%) patients died during the inpatient stay. Of them, 31 of 34 (90%) died from septic shock. One patient died of kidney failure, one of ventricular fibrillation, and one of small cell lung cancer progression.

**Table 2:** Patient demographics

Endpoint	THA (181)	TKA (165)
Mean age	67.6 ± 23.8	68.1 ± 31.1
Mean BMI	29.3 ± 4.2	29.1 ± 3.9
Women	82 (45%)	71 (43%)
Hospitalisation (days)	24 ± 11	23 ± 12
Primary infection	120 (66%)	92 (56%)
Re-infection	61 (34%)	73 (44%)

## Results synthesis

Patients who presented values of creatinine > 1.2 mg/dL evidenced a statistically significant greater risk of mortality (95% CI 0.0131 to 0.1132). Patients with a history of malignancy demonstrated evidenced a statistically significant greater risk of mortality (95% CI 0.1478 to 0.7497). Patients with dementia evidenced a statistically significant greater risk of mortality (95% CI 0.0398 to 0.3791). Patients with nicotine and alcohol abuse, diabetes mellitus, arterial hypertension, hereditary thrombophilia, hereditary haemorrhages, cerebrovascular diseases, coronary heart diseases, chronic obstructive pulmonary disease osteoporosis, liver cirrhosis, rheumatoid arthritis, acute dental infection did not demonstrate to influence in the in-hospital mortality rate in patients who underwent revision surgery for PJI of a THA or TKA. These results are shown in greater detail in Table 3.

**Table 3:** Comparison of survivorship and mortality rate according to patient comorbidity

Comorbidity	Survived (*n* = 312)	Died (*n* = 34)	OR	95% CI	Z	P
Nicotine abuse	44 (14%)	4 (12%)	1.2313	0.4136 to 3.6657	0.374	0.7
Alcohol abuse	12 (4%)	2 (6%)	0.6400	0.1371 to 2.9875	0.568	0.6
Renal insufficiency	70 (22%)	30 (88%)	0.0386	0.0131 to 0.1132	5.926	< 0.0001
Diabetes mellitus	86 (28%)	8 (24%)	1.2367	0.5390 to 2.8374	0.501	0.6
Hypertension	206 (66%)	24 (71%)	0.8097	0.3734 to 1.7559	0.534	0.6
Hereditary thrombophilia	2 (0.6%)	0 (0%)	0.5556	0.0261 to 11.8099	0.377	0.7
Genetics haemorrhagic diathesis	0 (0%)	0 (0%)	0.1104	0.0022 to 5.6525	1.097	0.3
Cerebrovascular diseases	28 (9%)	8 (24%)	0.3204	0.1326 to 0.7744	2.528	0.01
Coronary heart diseases	70 (22%)	10 (29%)	0.6942	0.3169 to 1.5208	0.912	0.4
History of malignancy	38 (12%)	10 (29%)	0.3328	0.1478 to 0.7497	2.655	0.008
COPD	48 (15%)	6 (18%)	0.8485	0.3335 to 2.1588	0.345	0.7
Osteoporosis	14 (5%)	0 (0%)	3.3518	0.1956 to 57.4362	0.834	0.4
Liver cirrhosis	6 (2%)	2 (6%)	0.3137	0.0608 to 1.6193	1.384	0.2
Dementia	8 (3%)	6 (18%)	0.1228	0.0398 to 0.3791	3.647	0.0003
Rheumatoid arthritis	16 (5%)	2 (6%)	0.8649	0.1902 to 3.9330	0.188	0.9
Acute dental infection	8 (3%)	0 (0%)	1.9261	0.1088 to 34.1025	0.447	0.7

Patient drug therapy did not impact the risk of mortality in PJI (Table 4).

**Table 4:** Comparison of survivorship and mortality rate according to patient medications

Compound	Survived (*n* = 312)	Died (*n* = 34)	OR	95% CI	Z	P
Coumarin	13 (4%)	4 (11%)	0.337	0.1036 to 1.0973	1.806	0.07
NOACs	37 (12%)	3 (9%)	1.378	0.5536 to 3.4343	0.69	0.5
Heparins	6 (2%)	1 (4%)	0.489	0.0877 to 2.7369	0.813	0.4
ACE inhibitors	144 (46%)	18 (52%)	0.813	0.4512 to 1.3704	0.848	0.4
Corticosteroids	28 (9%)	3 (7%)	1.378	0.5536 to 3.4343	0.69	0.5
Angiotensin-II-receptor antagonists	81 (26%)	11 (32%)	0.746	0.4043 to 1.3787	0.934	0.4

## Discussion

PJI are the second most common cause for revision surgery after arthroplasty procedures of the hip or knee joint, and thus a major cause for hospital re-admission of such patients. PJI account for 15.5% of ​​total hip arthroplasty and 14.5% of total ​​knee arthroplasty revision procedures [5]. In 2019, 287 people died as a result of a periprosthetic joint infection in Germany [18]. The mean patient age for PJI of the hip was 74 and 72 years in cases of PJI of the knee, respectively [5].

In the present analysis, the mean patient age was 69 years, similar to the previously reported demographic data [19]. On the one hand, the number of arthroplasty procedures has increased steadily in recent years (Fig. 1) and, on the other, the demographic development shows an ageing population [20], and, as a consequence, an increasing number of patients suffering from PJI must be expected.

Within the group of patients evaluated, most were males, aged between 61 and 80 years, and had a mean BMI of 29.5 kg/m^2^ and thus, by definition, pre-obese. In addition, many patients presented ASA scores greater than 2. The relative frequencies of pre-existing medical conditions in the cohort were compared with the prevalence given in the literature for the general population. This comparison showed that the analysed patients suffered more frequently from renal insufficiency, diabetes mellitus, rheumatoid arthritis and coronary heart disease [21–24]. The distribution of arterial hypertension, COPD, nicotine abuse and liver cirrhosis was not increased in our patients compared to the prevalence in the general population [25–28]. The present results refer exclusively to the data that could be collected from the available files or discharge reports of the patients. Since these documents may not always show all previous illnesses or the smoking and drinking behaviour of the patients, the completeness of the data cannot be guaranteed, and especially substance abuse may be underreported. We have to acknowledge further limitations of this study. Given the retrospective nature of this analysis, we were unable to standardize the treatment protocols. We must also be cautious about confounding factors in the context of PJI patients with multiple comorbidities. Also, the substantial mortality rate should be interpreted as a result of treating a patient population with major comorbidities, and not as a result of the surgical treatment. An increased mortality has been suggested for PJI revision procedures when compared to primary arthroplasty or non-septic revision procedures [29]. Natsuhara et al. reported a one-year weighted mortality rate of 4.22% after total hip PJI and five-year mortality of 21 s% [19]. In our data, the reported in-hospital mortality of 10% is significant and is very likely associated with the high rate of patients presented with bacteraemia. We found bloodstream infections in 17%, and systemic manifestations of PJI are associated with an increased in-hospital mortality [30]. Tokarski et al. identified positive blood cultures in 6%, and in cases with SIRS (three or four criteria) the mortality rate was 11%, compared to 0.6% (two criteria or less), respectively [30]. Accordingly, Shahi et al. reported that patients undergoing treatment for PJI have a twofold increase in in-hospital mortality, when compared to aseptic revisions [31]. Furthermore, the authors overserved that the in-hospital mortality rate of revision THA procedures for PJI was higher than those for interventional coronary procedures, kidney transplant or carotid surgery. Cancienne et al. evaluated a patient cohort after removal of an infected THA and found that 6% of the individuals deceased in the hospital setting [32]. These studies highlight the importance of infection control to reduce the increased mortality after total joint arthroplasty and the role of patient comorbidities. In our data, more than half of the patients who died suffered from renal insufficiency (88.2%) and/or arterial hypertension (70.6%). All other pre-existing conditions examined were present in less than half of the deceased. Given the different composition of the two groups (156 survivors and 19 deceased), a statement about a statistically significant association between previous illness and severity of the course of the disease is not appropriate. A recent report found similar results [33]. In that study, 679,010 total knee replacements implanted in England and Wales between 2003 and 2013 were followed for at least 12 months to ascertain the incidence of infection. The data were extracted from the National Prosthesis Register of the National Health Service (NHS). A total of 3,659 (0.5%) of these knee joint prostheses required revision procedures because of infection. These patients were compared with all the other patients who had no infection during this period. Almost twice as many patients who subsequently developed a PJI of the knee were male [33]. A meta-analysis from 2018 including 37 studies with a total of 2,470,827 patients evaluated PJIs in TKA and THA. Being male seemed to be a risk factor for PJI [34]. The German arthroplasty registry, on the other hand, shows that women and men had comparable hip or knee PJI [5]. Lenguerrand et al. identified a high ASA score and high BMI as risk factors. As in the present work, the patients were mostly between 60 and 79 years old, with comorbidities identified as risk factors being chronic pulmonary disease, diabetes mellitus, liver diseases, rheumatoid diseases and peripheral vascular diseases [33]. Risk factors for PJI included obesity, diabetes mellitus and chronic lung diseases. Furthermore, arterial hypertension, coagulopathies, congenital heart defects and neoplasia were reported as risk factors [34]. A meta-analysis from 2014 reported comparable results, in which a high BMI, diabetes mellitus, rheumatoid arthritis and coagulopathies were identified as significant risk factors. The non-significant risk factors included liver cirrhosis, alcohol abuse, arterial hypertension and dementia [35]. Also, a recent investigation identified a high BMI, rheumatoid arthritis and diabetes mellitus as risk factors for PJI [36]. The discussed studies support our assumptions about potential risk factors. Since only patients with PJI were included in the present work, no statistically significant or clinically relevant statements can be made on the basis of the analysed data beyond an assumption. To identify statistically significant risk factors, the patients examined in this study would have to be compared with all patients who received a knee or hip joint prosthesis at the University Hospital RWTH Aachen in the years 2007–2018, similar to the study design by Lenguerrand et al. [33]. In summary, male gender, obesity, a high ASA score, diabetes mellitus, rheumatoid arthritis, chronic lung diseases, coagulopathies, malignancies, and alcohol and nicotine abuse are risk factors for PJI. In contrast to the background of this data, individual preventive measures can be taken. In everyday clinical practice, it is important to identify these risk factors in every patient and optimize them, especially before elective surgery [37, 38]. The ASA score is indirectly influenced by lifestyle modifications (abstinence from alcohol and nicotine, nutritional advice) and by adjustment or optimization of treatment for pre-existing diseases and can therefore also be optimized preoperatively [38].

Given the significantly increased risk for PJI in diabetic individuals, surgical interventions in diabetes mellitus patients with hyperglycaemia should be avoided [39]. Screening of HbA1c to assess long-term blood glucose control with a target value of < 6.9% is a possible approach [37]. Shohat et al. suggested preoperative screening (HbA1c value) in all patients prior to elective surgery to also detect patients with hyperglycaemic blood sugar levels in previously unknown diabetes mellitus. This requirement is based on a study published in 2018, which found no significant difference in PJI or wound healing disorders in patients with known compared to patients with previously unknown diabetes mellitus [40]. Citak et al., in turn, refute the benefit of preoperative HbA1c screening by evidencing no difference between patients with a preoperative HbA1c of > 6.5% or < 6.5%. With regard to a suitable screening method to assess adequate preoperative blood sugar control to reduce the risk of infection, no uniform statement can be made at present. With regard to perioperative antibiotic prophylaxis because of the increased risk of bacterial mucosal infections in diabetics [41], Inabathula et al. found a reduction in PJI in the first 90 days after implantation with a 7-day postoperative oral antibiotic prophylaxis. 2,181 patients who underwent total hip or knee arthroplasty between 2011 and 2016 were examined. In patients treated after 2015, a seven-day postoperative antibiotic prophylaxis was implemented in addition to the perioperative antibiotic prophylaxis already carried out in previous years (one hour before the operation and 24 h after the operation). Among these, a significant reduction of PJI was observed for both hip arthroplasty and knee arthroplasty. Patients either received cefadroxil, clindamycin, or trimethoprim in combination with sulfamethoxazole. In another report, a reduction in PJI with prolonged antibiotic treatment after aseptic revisions of an arthroplasty was evidenced [42].

Urinary tract infections (UTI), skin and mucous membrane infections, acute arthritis and bacteraemia are well-described risk factors for PJI [43]. Preoperative antibiotic coverage can generally reduce the risk of postoperative infections after implantation of a primary hip or knee arthroplasty [44]. Elective interventions should be postponed until active infections have completely healed. Given the hematogenous dissemination of distant sources of infection, an infection of the prosthesis can also occur years after arthroplasty [43]. A uniform recommendation for general antibiotic prophylaxis in the context of primary prosthesis implantations does not yet exist. Alamanda and Springer examined preventive measures for periprosthetic infections in a report published in 2019. Articles found on this topic identified in PubMed and MEDLINE between 1990 and 2018 were used in this report to make recommendations for preventive measures. Perioperative antibiotic prophylaxis should therefore include a first-generation cephalosporin or, in the case of allergies to beta-lactam antibiotics, clindamycin or vancomycin. The time of administration should be as close as possible to the skin incision, but no later than one hour after the incision for cephalosporins and clindamycin and two hours after the incision for vancomycin [45]. Vancomycin should be reserved for patients with a history of MRSA. According to Almanda and Springer, prolonged antibiotic prophylaxis beyond 24 h after the operation is not recommended [45].

Sixty-two (68.1%) of the patients with a PJI of the hip were examined in the present analysis and 66 (78.6%) of the patients with PJI of the knee were overweight with a BMI of ≥ 25 kg/m^2^. Some studies described an association between obesity and an increased risk of poorer wound healing or PJI [43]. Prolonged operating times, an increased need for blood transfusions and pre-existing conditions that are often associated with obesity, including diabetes mellitus, are possible causes for this relationship. Iannotti et al. suggested that according to a consensus of the American Association of Hip and Knee Surgeons (AAHKS), a delay in joint arthroplasty should be considered from a BMI > 40 kg/m2, especially if other comorbidities are present [46].

In a comparison between non-smokers and active smokers or former smokers and active smokers, postoperative wound healing disorders were significantly more common in active smokers. Perioperative smoking cessation interventions can reduce surgical site infections [47]. In a meta-analysis on this topic published in 2012, a significantly lower risk of wound healing disorders was shown for patients who had stopped smoking three to four weeks before the operation compared to active smokers [48]. In the current study, no significant differences according to smoking habits were observed.

Additional limitations should be discussed. Information on the duration of intensive care unit stay is missing, representing an important limitation of the present study. Patients in whom only the liner was exchanged and those patients who required a complete prosthesis change were not considered separately, which may represent another important limitation, and should be addressed by future studies. Presumably, the mortality might be much higher when changing the prosthesis than when simply changing the inlay. However, these data were not available and further subgroup analyses were therefore not possible. Future studies should evaluate whether patients with PJI should receive postoperative intensive care admission as standard, or should undergo prolonged antibiotic treatment. Moreover, additional studies are required to establish the potential of minimally invasive surgical procedures (e.g. excision of the fistula) in the revision setting of PJI.

## Conclusion

Patients undergoing revision surgery for periprosthetic joint infection after total hip and knee arthroplasty show an increased in-hospital mortality in the presence of dementia, renal insufficiency, and history of malignancy. Based on the present results, further infection prevention and geriatric co-management strategies should be evaluated for patients undergoing revision arthroplasty of the hip.

## Data Availability

The data underlying this article are available at reasonable request to the senior author AD (arne.driessen@luisenhospital.de).
